# The Associations between Dietary Patterns and Short Sleep Duration in Polish Adults (LifeStyle Study)

**DOI:** 10.3390/ijerph15112497

**Published:** 2018-11-08

**Authors:** Jerzy Gębski, Marzena Jezewska-Zychowicz, Dominika Guzek, Monika Świątkowska, Dagmara Stangierska, Marta Plichta

**Affiliations:** 1 Department of Organization and Consumption Economics, Faculty of Human Nutrition and Consumer Sciences, Warsaw University of Life Sciences (SGGW-WULS), 159C Nowoursynowska Street, 02-787 Warsaw, Poland; marzena_jezewska_zychowicz@sggw.pl (M.J.-Z.); dominika_guzek@sggw.pl (D.G.); monika_swiatkowska@sggw.pl (M.Ś.); marta_plichta@sggw.pl (M.P.); 2 Section of Horticultural Economic, Faculty of Horticulture, Biotechnology and Landscape Architecture, Warsaw University of Life Sciences (SGGW-WULS), 159C Nowoursynowska Street, 02-787 Warsaw, Poland; dagmara_stangierska@sggw.pl

**Keywords:** dietary patterns, short sleep duration, adults, principal component analysis

## Abstract

Short sleep duration appears to put adults at risk of excessive energy intake and obesity; less is known specifically about how sleep quantity relates to dietary patterns. Therefore, the aim of this study was to assess the associations of dietary patterns (DPs) with short sleep duration. The data were collected in November 2016 through a cross-sectional quantitative survey among 972 Polish adults with both normal weight and excessive weight. Principal components analysis (PCA) was conducted to derive DPs. Logistic regression analysis was used to verify associations between short sleep duration (independent variable) and DPs (dependent variables). Five DPs (‘Fast foods & sweets’—FF&S; ‘Meat & meat products’—M&MP; ‘Fruit & vegetable’—F&V; ‘Wholemeal food’—WF; and ‘Fruit & vegetable juices’—F&VJ) were identified. Adults whose diet was based both on fruit and vegetables (OR 0.62, 95% CI: 0.44–0.88; *p* < 0.01) and on fast food and sweets (OR 0.63, 95% CI: 0.44–0.99; *p* < 0.01) were less likely to be short sleepers on weekdays. On weekdays short sleep duration was associated with smaller odds of FF&S both for men (OR 0.58, 95% CI: 0.33–0.97; *p* < 0.05) and for women (OR 0.61, 95% CI: 0.38–0.98; *p* < 0.05), and with smaller odds of F&V (OR 0.34, 95% CI: 0.20–0.58; *p* < 0.001) for men. Short sleep duration on weekend days was associated with a smaller odds of F&VJ (OR 0.56, 95% CI: 0.30–0.96; *p* < 0.05- only in crude model) and higher odds of F&V (OR 1.70, 95% CI: 1.02–3.11; *p* < 0.05) for women, and with a smaller odds of FF&S (OR 0.37, 95% CI: 0.17–0.79; *p* < 0.05) for men. The number of significant associations between dietary patterns and sleep duration was higher among people with normal weight and overweight compared to those with BMI ≥ 30. We found that both short sleep duration on weekdays and weekend days was associated with some dietary patterns including both healthy and unhealthy DPs. Gender significantly differed these relationships in groups with normal and excessive weight. Findings of the study confirmed the potential effectiveness of combined use of sleep duration and dietary patterns in the development of recommendations for prevention of obesity. Future studies are called for to evaluate these links using dietary patterns identified according to food intake.

## 1. Introduction

The role of diet and exercise has dominated previous research on the etiology of obesity [1]. However more recently, sleep has been identified as a health related behavior that may play an essential role in conditioning obesity as well [2]. Although some empirical evidence indicates significant associations between habitual sleep duration (both short and long sleep) and obesity [3,4,5,6,7], there are also studies that do not confirm such a relationship [8,9]. Thus, short sleep duration is still questionable as a risk factor for obesity [9]. Moreover, the results of previous studies confirmed positive associations of sleep duration with the occurrence of other diseases, i.e., cardiovascular disease [10,11], type 2 diabetes [11], and hypertension [12]. The adverse health outcomes in children and adults are associated with both too little and too much sleep [6,13,14,15,16].

Previous studies on the determinants of sleep duration identified socioeconomic factors (low income levels, living alone, and low self-perceived social status) [17], environmental factors (temperature, noise, light, comfort level of the bed, and electronic distractions), and lifestyle factors (living in a disadvantaged neighborhood, screen use, marital status, family conflict, and financial problems) as contributing to poor sleep both in children and adults [4,18,19]. Dietary patterns should be also perceived as important lifestyle factor when short sleep duration is considered [20,21]. Moreover, the impact of occupational factors like shift work and long work hours on sleep duration has been highlighted [4,22]. In addition, some medical conditions, medications, and sleep disorders like sleep apnea affect duration and quality of sleep [23]. Many of these factors are also associated with obesity [20,23,24].

Despite the general agreement across studies that there is an association between sleep duration and obesity [3,25], there is some notable inconsistency regarding the nature of this relationship, inter alia, after considering age [26,27]. Over the last decades, the relationship of diet and sleep duration was also confirmed. Studies have shown that short sleepers have higher energy intakes, particularly from fat [28,29] and snacks [30,31] compared to respondents reporting 7–8 h of sleep. Those who slept less than 7 h a night consumed a smaller variety of foods with lower protein, carbohydrate, and fiber content than the normal sleepers [30]. However, evidence relating to nutrient intake in relation to sleep duration is contradictory. For example, it has been shown that short sleepers consume both foods with lower fat [32] and foods with higher fat content [28,29]. Similar inconsistencies have been observed in regards to carbohydrate intake. Both higher carbohydrate intake [33,34] and lower carbohydrate intake [32] have been observed for short sleepers. Besides, the type of carbohydrate seems to be more important than its quantity in mediating the association of sleep duration and body mass index (BMI) [34]. Dietary behaviors also appear to be related to sleep duration. It was observed that short sleep duration was associated with irregular eating [20,21], unbalanced food variety [20], trying to eat less [20], snacking between meals, and eating out [21].

Differences in the dietary behaviors between short sleepers and others may result from the fact that short sleep duration as well as disrupted sleep lead to emotional stress, impaired decision-making, and increased reward sensitivity to calorie-dense foods [24], especially among obese individuals [23]. An evidence of links between sleep quality and diet is even larger compared to sleep duration [34,35,36,37,38]. Poor sleep quality was associated with high intake of confectionary and noodles, while a high intake of fish and vegetables was associated with good sleep quality [34]. Skipping breakfast and eating irregularly were also strongly associated with poor sleep quality [34].

The available evidence of the associations between diet and sleep quality is quite strong, but still limited for sleep duration. Mechanisms mediating the associations between duration and quality of sleep and dietary intake are likely to be multifactorial and include differences in the appetite-related hormones leptin and ghrelin, hedonic pathways, extended hours for intake, and altered time of intake [39]. Although these mechanisms have not been analyzed in our study, their effects have probably been reflected in the observed associations between short sleep duration and dietary patterns.

In general, the results from studies investigating the link between sleep duration and foods support the link between short sleep duration and lower intakes of healthy foods [39]. Most of these studies concern particular dietary characteristics, that is, nutrients and/or foods, not dietary patterns (DPs). DPs describe the overall diet including the foods, food groups, and nutrients, their combination and variety, and the frequency and quantity with which they are habitually consumed [40,41,42,43]. It seems that the links between dietary patterns and the length of sleep may be of great importance in explaining adverse health consequences, but these studies still remain sparse. Therefore, it is unknown whether or how dietary patterns are associated with short sleep duration. To the best of our knowledge there have been no studies using the dietary patterns (DPs) where links between the diet and sleep duration were investigated.

In terms of prevention, sleeping habitually 7 and 8 h a night may be optimal for the health of an adult [44]. People reporting sleeping 6 h or less a night should be regarded as a group with higher risk for all-cause mortality [10]. Although the impact of short sleep duration on dietary intake seems to be rather small [39], it may contribute to an increased risk of obesity in people declaring chronic short sleep [23]. In the previous studies, the associations of dietary patterns with gender and BMI have been recognized quite well [45,46], but those with sleep duration have not been reported. Gender differences in the risk of diseases related to duration of sleep have also been reported [47,48,49], but not in relation to dietary patterns. Thus, to address these gaps in the evidence, the aim of this study was to assess the association of dietary patterns with short sleep duration, derived from a self-reported questionnaire carried out in a Polish sample consisting of people with both normal weight and excessive weight. Gender and BMI have been included as variables that can modify this relationship. The diagnosis of this relationship is justified from the perspective of obesity prevention.

## 2. Materials and Methods

### 2.1. Ethical Approval

The study was approved by the Ethics Committee of the Faculty of Human Nutrition and Consumer Science, Warsaw University of Life Sciences, in Poland on the 27th of June 2016 (Resolution No. 01/2016). The study was conducted in compliance with the guideline statements of the Declaration of Helsinki. Informed consent to participate in the study was collected from participants.

### 2.2. Study Design and Sample Collection

The data were collected in November 2016 through a cross-sectional quantitative survey under the LifeStyle Study, in which frequency of consumption of selected food groups, self-reported physical activity in leisure time and work/school time, sedentary behaviors including reading books and newspapers, watching TV and computer use, and self-reported sleep duration were measured. According to the study design, recruitment and data collection were conducted by a research agency—ARC Market and Opinion. Details regarding recruitment protocol and methodology were previously reported [50]. In brief, 1007 adults aged 21–65 were recruited from a panel of approximately 55,000 registered adults. The sampling was applied to ensure the sample is representative for the Polish population. The computer-assisted web interviewing (CAWI) technique was used to collect all data. A questionnaire script provided on a webpage increases the sense of anonymity and gives an opportunity to participate in the study at a time convenient for the respondent which in turn allows for the collection of more accurate data. The criterion of inclusion in the sample was normal weight (BMI = 18.5–24.9 kg/m^2^), overweight (BMI = 25–29.9 kg/m^2^), or obese (BMI ≥ 30 kg/m^2^). Thirty-five participants were excluded from the analyses due to BMI lower than 18.5 kg/m^2^. Thus, the total sample consisted of 972 people with BMI ≥18.5 kg/m^2^.

### 2.3. Eating Habits

The Beliefs and Eating Habits Questionnaire (KomPAN), developed and validated by the Commission of Behavioral Determinants of Nutrition from the Polish Academy of Sciences [51], was used to assess the frequency of consumption of selected food groups, including wholemeal bread; wholemeal pasta and groats; fermented milk drinks; cheeses (including melted cheese and blue cheese); cured meats and sausages; red meat; white meat; fried foods; fruits; vegetables; vegetable juices; fruit juices; fizzy drinks; meals or snacks such as burgers, pizza, chicken, fries; sweets and cakes; and crisps and other salty snacks. All participants were asked to record their habitual intake frequency for each food group within the last year according to the following categories. 1—never; 2—less often than once a month, 3—from 1 to 3 times a month; 4—once a week, 5—several times a week; 6—once a day; 7—several times a day.

### 2.4. Sleep Duration

Sleep duration was recorded separately for weekdays and weekend days on a 3-point scale ranging from 1 to 6 h or less a night (short sleep); 2–7 or 8 h a night (optimal sleep); and 3–9 or more hours a night (long sleep). The scale implemented to assess the duration of sleep reflects previous studies [32,52,53,54,55,56].

### 2.5. Sociodemographic Variables

The questionnaire collected information about sociodemographic characteristics of the sample including gender, age, education, and place of residence. Body Mass Index (BMI) was calculated using self-reported weight and height and categorized according to International Obesity Task Force (IOTF) standards [56].

### 2.6. Statistical Analysis

A principal components factor analysis (PCA) was conducted to derive dietary patterns based on the frequency of eating products from sixteen food groups. Details regarding the derived dietary patterns were previously reported [50]. In brief, the factors were rotated by an orthogonal (Varimax) transformation. The number of factors was based on the following criteria, components with an eigenvalue of 1 and higher, scree plot test and the interpretability of the factors. The eigenvalues signify the amount of variance explained by each of the factors. KMO value was 0.781, and Bartlett’s test had a significance of *p* < 0.0001 [57]. Five dietary patterns (factors) were derived: ‘Fruit & vegetable’, ‘Wholemeal food’, ‘Fast foods & sweets’, ‘Fruit & vegetable juices’, and ‘Meat & meat products’, accounting for 66.2% of total variance.

Based on the tertile distribution participants were divided into three categories within each pattern (bottom, middle, and upper tertile). The upper tertile (T3) represents the greatest adherence and the bottom tertile (T1) represents the lowest adherence to the DPs.

Logistic regression analysis was used to verify associations between both variables expressing sleep duration (independent variables) and DPs (dependent variables). The variables describing sleep duration were as follows, short sleep and longer than short sleep separately for weekdays and weekend days.

Odds ratios (ORs) represented the chances of the adherence to upper tertiles of each DP. The reference groups (OR = 1.00) were those who represented the bottom tertile of each DP. Two models were created: one was crude and the other was adjusted for age (as continuous variable), education, and place of residence (as categorical variables) [6,30,58]. Wald’s test was used to assess the significance of ORs. Tests of linear trend across increasing tertiles of DPs adherence (for ORs) were calculated for sleeping time. A *p*-value of ≤ 0.05 was considered as significant for all tests. All analyses were carried out applying sample weights to adjust for nonresponse and missing data. All analyses were performed using SAS 9.4. software (SAS Institute, Cary, NC, USA).

## 3. Results

### 3.1. Sample Characteristics

The sample consisted of 972 participants (499 women and 473 men) aged 21 to 65 years old. Details concerning sociodemographic characteristics of the study sample are displayed in Table 1.

The sample included 484 adults with normal weight (18.5 kg/m^2^ ≤ BMI < 25 kg/m^2^) and 488 participants were overweight or obese (BMI ≥ 25 kg/m^2^). Significantly more men than women were either overweight or obese. The normal weight characterized almost twice as many people with higher education compared to those with secondary education. The largest number of people with normal weight were 21 to 34 years old, while among those with BMI ≥ 25, a larger proportion were aged from 55 to 65 years old (Table 1).

### 3.2. Dietary Patterns

The factor-loading matrix for the DPs identified by principal component analysis (PCA) was previously presented in detail [50]. In brief, the ‘Fast foods & sweets’ pattern (FF&S) comprised crisps and other salty snacks (0.824), meals or snacks such as burgers, pizza, chicken, fries (0.756), sweets and cakes (0.702), and fizzy drinks (0.633); the ‘Meat & meat products’ pattern (M&MP) was composed of red meat (pork, beef, and venison) (0.783), white meat (poultry and turkey) (0.748), cured meats and sausages (0.696), and fried foods (0.551); the ‘Fruit & vegetable’ pattern (F&V) was composed of fruits (0.825) and vegetables (0.764); the ‘Wholemeal food’ pattern (WF) was comprised of wholemeal pasta, groats (0.839), and wholemeal bread (0.763); and the ‘Fruit & vegetable juices’ pattern (F&VJ) was composed of vegetable juices (0.830) and fruit juices (0.799). The mean standardized values in individual tertiles are significantly different within DPs [50].

### 3.3. Sleep Duration

**Figure 1:**
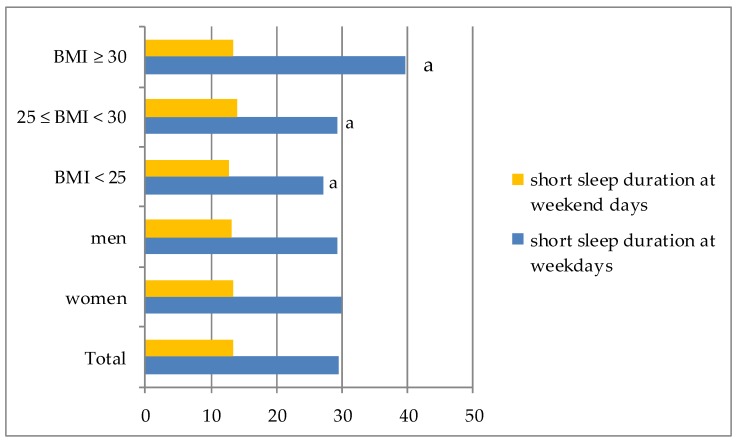
Short sleep duration in the sample by gender and BMI separately. ^a^ significantly different according to BMI (*p*-value ≤ 0.05, Chi-square test).

Almost 30% of the sample was short sleepers on weekdays, while only 13.3% of participants were short sleepers at the weekend. There were no significant differences in the percentage of short sleepers both on weekdays and on weekend days in groups identified according to gender. In short sleepers the majority had a BMI ≥ 30 and the minority had normal weight (*p* < 0.05) (Figure 1).

**Figure 2:**
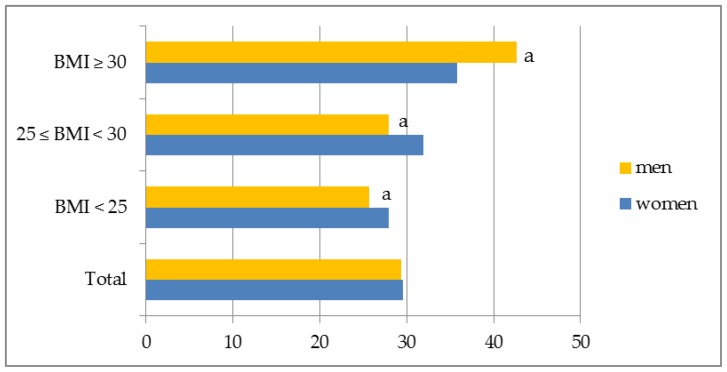
Short sleep duration on weekdays in the sample by gender and BMI. ^a^ significantly different according to BMI (*p*-value ≤ 0.05, Chi-square test).

**Figure 3:**
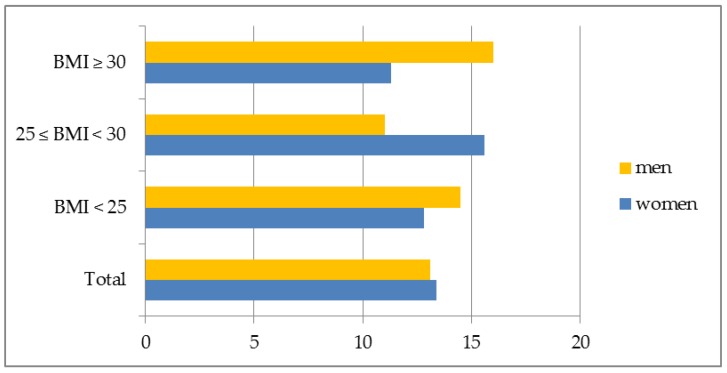
Short sleep duration on weekend days in the sample by gender and BMI.

There were no significant differences in the percentage of female short sleepers on weekdays in groups identified according to BMI. In men with BMI ≥ 30 the majority was short sleepers, whereas the smallest number of men with normal weight belonged to this category (*p* < 0.05) (Figure 2). On weekend days, no significant differences among short sleepers in regard to BMI and gender were observed (Figure 3).

### 3.4. Dietary Patterns versus Sleep Duration

The results of both crude and adjusted models have demonstrated that people who consumed fruit and vegetables most often (the upper tertile of F&V) were less likely to be short sleepers on weekdays. There were no other significant associations of dietary patterns and short sleep duration on weekdays. Moreover, in the adjusted model a significant association was found between the upper tertile of FF&S and short sleep duration on weekend days (Table 2).

The results from both models have shown that both women and men were less likely to be short sleepers on weekdays in the upper tertile of the ‘Fast foods & sweets’ pattern. Moreover, men were also less likely to be short sleepers on weekend days in the upper tertile of FF&S pattern. Only men who ate fruit and vegetables most often (the upper tertile of F&V) were less likely to be short sleepers on weekdays. Women were more likely to be short sleepers on weekdays in the upper tertile of WF (crude model).

People in the upper tertile of the ‘Fast foods & sweets’ pattern were less likely to be short sleepers on weekend days. Moreover, on weekend days women were more likely be short sleepers in the upper tertile of F&V and were less likely be short sleepers in the upper tertile of F&VJ. However, in the adjusted model, significant associations between the upper tertile of F&VJ pattern and short sleep duration in women were not confirmed (Table 2).

People with normal weight were less likely to be short sleepers in the upper tertile of the ‘Fast foods & sweets’ pattern both on weekdays and weekend days. People who ate fruit and vegetables most often (the upper tertile of F&V) were less likely to be short sleepers only on weekdays. However, those who ate wholemeal food most often (the upper tertile of WF) were more likely to be short sleepers on weekdays. These results were consistent in both crude and adjusted models (Table 2). In the upper tertile of FF&S, women with normal weight were less likely to be short sleepers on weekdays and weekend days, while men were less likely to sleep so little only on weekend days. Both crude and adjusted models have shown that men were less likely to be short sleepers on weekdays in the upper tertile of ‘Fruit & vegetables’ pattern. Women with normal weight were more likely to be short sleepers in the upper tertile of ‘Wholemeal’ pattern both on weekdays and weekend days (Table 3).

Overweight people were less likely to be short sleepers in the upper tertile of FF&S and FV patterns on weekdays. Simultaneously, on weekend days they were less likely to be short sleepers in the upper tertile of WP pattern and more likely to be short sleepers in the upper tertile of M&MP. These results were consistent in both crude and adjusted models (Table 2). In overweight women, no significant associations of dietary patterns with short sleep duration were found. The results of the adjusted models have shown that overweight men were less likely to be short sleepers on weekdays in the upper tertiles of ‘Fruit & vegetables’ and ‘Fast food & sweets’ pattern. However, they were more likely to be short sleepers on weekdays when representing the upper tertile of M&MP pattern (Table 3).

Obese people were less likely to be short sleepers in the upper tertile of FV pattern on weekdays (Table 2). In obese women, a significant association between the F&V pattern and short sleep duration on weekend days was found, while obese men who ate fruits and vegetables most often (the upper tertile of F&V) were less likely be short sleepers on weekdays (Table 3).

## 4. Discussion

Our findings support the results of previous studies indicating lower intakes of fruits and vegetables among short sleepers [16,21,59,60,61]. Although people in the upper tertile of the ‘Fruit & vegetable’ pattern were less likely to be short sleepers, this association only applies to the weekdays. This may be related to work activity [21], allowing limited time for eating [62], no easy access to fruit and vegetable at worksites [63], and low popularity of eating outside home [64]. In turn, missing at least one main meal per day or irregular eating resulted in eating less fruits and vegetables [65,66]. In such a situation, improving fruit and vegetable accessibility in worksites might improve their intake in adults [67]. It can be assumed that on weekend days the availability of fruits and vegetables increases due to longer stay at home and more regular consumption of meals. In addition, vegetables and especially fruits are increasingly eaten between meals as snacks [68,69] which could have a significant impact on the declared frequency of eating this food on weekend days.

We found that on weekdays only men in the upper tertile of the ‘Fruit & vegetable’ pattern were less likely to be short sleepers, this was found for both men with normal weight and excessive weight. Similarly, in the Japanese sample it was observed that male short sleepers (<6 h) reported consuming fewer vegetables than normal sleepers (6–9 h) [21]. An explanation of our finding concerning the consumption of fruits and vegetables on weekdays in men may involve the arguments presented previously [63,64], whereas in women who are more concerned about their health [70,71], higher intake of these may be favored regardless of working conditions.

On the other hand, women who often ate fruit and vegetables (the upper tertile of F&V) were more likely to declare short sleep duration on weekend days. Our results in women are not entirely consistent with previous data which support the associations between short sleep duration and a lower intake of fruits [33,49,60]. However, there are also studies that did not confirm this relationship in women [72].

On weekend days BMI did not differentiate the chances to be short sleepers when representing the upper tertile of F&V pattern. However, on weekdays, all groups identified by BMI were less likely to be short sleepers when frequent consumption of fruit and vegetable was reported. Regardless of BMI, only men in the upper tertile of F&V were less likely to be short sleepers on weekdays. Previous studies showed that short sleep duration (6 h or less) was associated with an increased risk of obesity only in men [73,74]. Although Charlton et al. [75] showed that overweight men were less likely to consume high servings of both fruit and vegetables and to meet dietary targets for fruit and vegetables compared to men of normal weight, our findings concerning weekdays, did not confirm the differences between groups identified by BMI, also in men.

Our findings have demonstrated that the participants with normal weight in the upper tertile of WF pattern were more likely to be short sleepers on weekdays. Moreover, only women with normal weight who often ate wholemeal food (the upper tertile of WF pattern) were more likely to be short sleepers, both on weekdays and weekend days. Thus, the coexistence of both healthy (frequent consumption of wholemeal food) and unhealthy (short sleep duration) components of lifestyle was observed in those women. Results from previous studies support mostly the relation between short sleep duration and unhealthy diet [39,76,77], lower intakes of healthy foods, mainly fruit and vegetables [21,33,59,60], also dietary fiber [32,33], whole grains, and beans [33]. Thus, our study has showed an inverse association, namely that people with normal weight who ate wholemeal food were often more likely to be short sleepers on weekdays, however, gender differences were observed. This relationship was noticed only in women.

In previous studies the shift from meals to snacks with short sleep duration was observed [21,28,49,78]. Moreover, evidence suggests that short sleepers have highly palatable snacks at night more frequently [39]. However, Kant & Graubard [52] observed that the total number of eating episodes and snacking was not related to sleep duration. While some previous studies have indicated an increase in snacking among short sleepers [28,39,49], our study revealed an inverse association between short sleep duration and the ‘Fast food & sweets’ pattern which includes foods mostly eaten between meals. Namely, those consuming fast food, sweets, and fizzy drinks less frequently were more likely to be short sleepers, both on weekdays and weekend days. On weekend days such associations were not observed in women and in people with BMI ≥ 25. Nevertheless, these results need to be confirmed in further studies in which both the frequency of consumption and the quantity of eaten fast food, sweets and fizzy drinks would be assessed.

While quite similar results on the links between length of sleep and diet were obtained in previous studies, some inconsistencies are also observed which may have from many reasons. Firstly, a great variability in the cutoffs was used to define short sleep duration. The most common cutoffs are less than 6 h [20,21,33,61], 6 h and below [32,52,53,54], and less than 7 h [49,59], which often represent similar groups. Secondly, the dietary assessment methods were different, i.e., either the absolute [32,52] or relative [52] intake of nutrients or foods [21,59,60,61] were assessed. Finally different samples were evaluated.

Despite the methodological differences, the vast majority of studies showed a link between sleep duration and diet regardless of whether it was evaluated by the amount of food or nutrients and the frequency of eating. Our research confirmed that the identified dietary patterns were also significantly associated with short sleep duration. Although some of the previous results have been confirmed, inconsistent results were also obtained. Short sleep duration was found to be associated with both unhealthy and healthy dietary patterns (‘Fast food & sweets’, ‘Meat & meat products’, ‘Fruit & vegetables’, and “Whole foods’, respectively). Nevertheless, the nature of some associations of short sleep duration with healthy dietary patterns were reversed, i.e., a link between short sleep duration and more frequent consumption of wholemeal food among people with normal weight but also a link between short sleep duration and less often consumed fast-foods, sweets, and fizzy drinks. Our findings have demonstrated that associations between DPs and sleep duration were varied, both due to gender and BMI. However, further research is still required—it should involve the dietary patterns instead of some foods or nutrients in groups identified by gender and BMI.

The strength of our results is a relatively large representative sample of Polish population. Nevertheless, the findings have some limitations. The cross-sectional design of the present study and data collection at a single point in time did not allow conclusions to be drawn about causality, but only on the associations between short sleep duration and dietary patterns. Future research should include a greater emphasis on longitudinal studies in order to examine how sleep duration and BMI vary within an individual over time. The use of the questionnaire is also limited due to overestimation of the consumption of certain foods when FFQs is used. We have chosen FFQ because we aimed to see predominantly ‘healthy’ and ‘unhealthy’ dietary patterns, rather than exact amount of foods. Other limitation relates to the potential biases that may occur when self-reported data are analyzed. Thus, the possibility of social desirability bias should be taken into account [79]. Moreover, sleep duration was measured by a single self-reported item, which may entail some interpretative difficulties, especially because sleep was described in whole hours without the possibility to indicate exact minutes. More reliable results could be achieved through objective means (e.g., actigraphy) or other prospective self-reports (e.g., sleep diaries). However, sleep diaries, actigraphy, and polysomnography from some large population and small-scale investigations have shown high correlations between subjective estimates of sleep duration and the more direct assessments [80,81,82]. A single self-reported sleep duration may be a crucial variable when thinking about interventions focused on health, since dieticians typically intervene based on such information. Our study focused on the associations between short sleep duration and dietary patterns with adjustment only on sociodemographic variables. Further research should include other factors influencing both sleep duration and dietary patterns, among them emotional distress [23].

The observed links between both healthy as well as unhealthy DPs and short sleep duration are of great importance for public health in Poland. Although findings should not be generalized to populations with different cultural backgrounds, our study provides an interesting insight into dietary patterns and their association with sleep duration. If the results of our study were confirmed in further studies this would highlight the importance of disseminating scientific evidence focusing on the relationship between sleep duration and diet into practical messages that help the public to improve their well-being and prevent chronic diseases. This would include making different groups aware of the associations between sleep and diet by providing more information on sleep in national dietary guidelines to enhance healthy lifestyle recommendations. Moreover, this information can be provided in hospitals and medical schools to educate healthcare professionals to develop effective weight loss programs and other programs targeting improvement in overall health.

## 5. Conclusions

The study revealed the coexistence of the associations between both healthy as well as unhealthy dietary patterns and short sleep duration. People who ate fruits and vegetables more frequently (the upper tertile of F&V) as well as those who consumed fast-food, sweets, and fizzy drinks (the upper tertile of FF&) were less likely to be short sleepers. The differences in the relationships between dietary patterns and short sleep duration were observed on weekdays and weekend days, both in women and men. Moreover, BMI determined the relationship between dietary patterns and short sleep duration differently in men and in women. The number of significant associations between both healthy and unhealthy dietary patterns and sleep duration were higher among people with normal weight and overweight compared to those with BMI ≥ 30. Among obese people, and actually among obese men, only the relationship between sleep duration and F&V pattern was confirmed. The findings of our study confirmed the potential effectiveness of combined use of both factors (diet and sleep duration) in the development of recommendations aimed at the prevention of obesity. However, future studies are called for to evaluate the links using dietary patterns identified with regard to food intake.

## Figures and Tables

**Table 1:** Study sample characteristics.

Variables		Total Sample		18.5 ≤ BMI < 25		25 ≤ BMI < 30		BMI ≥ 30	
		N = 972	100%	N = 484	100%	N = 360	100%	N = 128	100%
Gender *	Female	499	51.3	305	63.0	141	39.2	53	41.4
	Male	473	48.7	179	37.0	219	60.8	75	58.6
Age *	21–34 years old	346	35.6	227	46.9	101	28.1	18	14.1
	35–44 years old	228	23.5	116	24.0	86	23.9	26	20.3
	45–54 years old	131	13.5	49	10.1	48	13.3	34	26.5
	55–65 years old	267	27.4	92	19.0	125	34.7	50	39.1
Place of residence	City ≤ 50,000 residents	195	20.1	88	18.2	81	22.5	26	20.3
	City > 50,000 residents	521	53.6	269	26.2	190	52.8	62	48.4
	Rural area	256	26.3	127	55.6	89	24.7	40	31.3
Education *	Upper secondary and lower	388	39.9	156	32.2	164	45.6	68	53.1
	Higher	584	60.1	328	67.8	196	54.4	60	46.9

N—number of participants; * Significant at *p* < 0.0001.

**Table 2:** Adjusted associations between dietary patterns and sleep duration by gender and BMI (adjusted odds ratios with 95% confidence intervals).

Dietary Patterns	Short Sleep Duration	Model	Total Sample		Women		Men		18.5 ≤ BMI < 25		25 ≤ BMI < 30		BMI >= 30	
			Upper Tertile	*p*	Upper Tertile	*p*	Upper Tertile	*p*	Upper Tertile	*p*	Upper Tertile	*p*	Upper Tertile	*p*
Fast foods & sweets	at weekdays	Crude ^a^	0.68 (0.48; 0.95)	*	0.67 (0.43; 0.95)	*	0.61 (0.32 0.95)	*	0.64 (0.39; 0.96)	*	0.48 (0.26; 0.88)	*	1.15 (0.44; 3.01	
(ref. bottom tertile)		Adjusted ^b^	0.63 (0.44; 0.90	**	0.61 (0.38; 0.98)	*	0.58 (0.33; 0.97)	*	0.55 (0.32; 0.93)	*	0.45 (0.23; 0.85)	*	1.68 (0.59; 4.79)	
	at weekend	Crude	0.69 (0.45; 1.07)		0.79 (0.44; 1.39)		0.44 (0.21; 0.92)	*	0.51 (0.27; 0.96)	*	0.63 (0.28; 1.41)		1.23 (0.29; 5.16)	
		Adjusted	0.61 (0.38; 0.96)	*	0.70 (0.38; 1.28)		0.37 (0.17; 0.79)	*	0.42 (0.22; 0.82)	*	0.57 (0.24; 1.36)		1.23 (0.26; 5.78)	
Fruit & vegetable	at weekdays	Crude	0.61 (0.44; 0.86)	**	0.99 (0.62; 1.57)		0.33 (0.20; 0.56)	***	0.59 (0.35; 0.97)	*	0.50 (0.28; 0.89)	*	0.37 (0.13; 0.96)	*
(ref. bottom tertile)		Adjusted	0.62 (0.44; 0.88)	**	1.01 (0.63; 1.61)		0.34 (0.20; 0.58)	***	0.61 (0.36; 0.98)	*	0.50 (0.27; 0.92)	*	0.21 (0.06; 0.67)	*
	at weekend	Crude	1.23 (0.79; 1.92)		1.71 (1.04; 3.09)	*	0.88 (0.44; 1.76)		1.16 (0.61; 2.19)		1.46 (0.66; 3.21)		0.15 (0.03; 0.76)	
		Adjusted	1.25 (0.80; 1.97)		1.70 (1.02; 3.11)	*	0.95 (0.46; 1.93)		1.22 (0.63; 2.33)		1.48 (0.64; 3.40)		0.16 (0.03; 0.91)	
Wholemeal food	at weekdays	Crude	1.25 (0.89; 1.76)		1.36 (0.85; 2.16)	*	1.14 (0.67; 1.95)		1.82 (1.07; 3.08)	*	0.57 (0.31; 1.04)		1.66 (0.65; 4.22)	
(ref. bottom tertile)		Adjusted	1.22 (0.86; 1.70)		1.35 (0.85; 2.16)		1.11 (0.65; 1.90)		1.80 (1.05; 3.10)	*	0.57 (0.31; 1.05)		1.45 (0.55; 3.83)	
	at weekend	Crude	0.90 (0.58; 1.40)		1.16 (0.64; 2.12)		0.65 (0.33; 1.26)		1.46 (0.74; 2.88)		0.43 (0.20; 0.92)	*	0.84 (0.23; 3.03)	
		Adjusted	0.84 (0.54; 1.37)		1.14 (0.62; 2.09)		0.58 (0.29; 1.14)		1.39 (0.70; 2.77)		0.37 (0.16; 0.83)	*	0.80 (0.21; 3.00)	
Fruit &vegetable juices (ref. bottom tertile)	at weekdays	Crude	0.97 (0.69; 1.36)		0.87 (0.54; 1.38)		1.15 (0.69; 1.90)		0.80 (0.48; 1.32)		1.41 (0.80; 2.48)		0.63 (0.23; 1.70)	
		Adjusted	0.99 (0.71; 1.40)		0.91 (0.57; 1.46)		1.14 (0.69; 1.30)		0.79 (0.47; 1.32)		1.48 (0.83; 2.66)		0.62 (0.21; 1.83)	
	at weekend	Crude	0.69 (0.44; 1.08)		0.56 (0.30; 0.96)	*	0.92 (0.48; 1.76)		0.65 (0.34; 1.26)		0.74 (0.34; 1.59)		0.55 (0.13; 2.26)	
		Adjusted	0.70 (0.44; 1.11)		0.62 (0.33; 1.19)		0.86 (0.44; 1.70)		0.67 (0.34; 1.31)		0.79 (0.35; 1.70)		0.45 (0.10; 2.11)	
Meat & meat products (ref. bottom tertile)	at weekdays	Crude	1.13 (0.80; 1.59)		1.02 (0.63; 1.65)		1.41 (0.85; 2.34)		0.98 (0.58; 1.63)		2.22 (1.23; 4.00)		0.47 (0.16; 1.39)	
		Adjusted	1.14 (0.81; 1.61)		1.03 (0.64; 1.68)		1.39 (0.84; 2.30)		0.96 (0.57; 1.62)		2.28 (1.24; 4.19)		0.35 (0.11; 1.15)	
	at weekend	Crude	0.90 (0.57; 1.43)		0.87 (0.46; 1.62)		0.97 (0.48; 1.96)		0.87 (0.44; 1.71)		1.25 (0.56; 2.77)	**	0.14 (0.02; 1.29)	
		Adjusted	0.88 (0.55; 1.40)		0.82 (0.43; 1.55)		0.89 (0.43; 1.84)		0.86 (0.43; 1.72)		1.08 (0.47; 2.48)	**	0.13 (0.01; 1.25)	

^a^ Odds ratio unadjusted ^b^ Odds ratio adjusted for age, education and place of residence; Statistically significant: * *p* < 0.05; ** *p* < 0.01; *** *p* < 0.001 (Wald’s test).

**Table 3:** Adjusted associations between dietary patterns and sleep duration within BMI groups by gender (adjusted odds ratios with 95% confidence intervals).

Dietary Patterns	Short Sleep Duration	Model	18.5 ≤ BMI < 25				25 ≤ BMI < 30				BMI ≥ 30			
			Women		Men		Women		Men		Women	Men		
			Upper Tertile	*p*	Upper Tertile	*p*	Upper Tertile	*p*	Upper Tertile	*p*	Upper Tertile	*p*	Upper Tertile	*p*
Fast foods & sweets	at weekdays	Crude ^a^	0.58 (0.31; 0.98)	*	0.60 (0.22; 1.65)		0.77 (0.31; 1.89)		0.26 (0.10; 0.67)	**	1.22 (0.25; 5.90)		1.23 (0.30; 5.06)	
(ref. bottom tertile)		Adjusted ^b^	0.48 (0.24; 0.93)	*	0.58 (0.21; 1.65)		0.82 (0.32; 2.14)		0.23 (0.08; 0.63)	**	2.07 (0.35; 12.13)		1.74 (0.36; 8.39)	
	at weekend	Crude	0.55 (0.25; 1.20)		0.22 (0.04; 0.98)	*	1.21 (0.35; 4.19)		0.27 (0.07; 1.07)		4.83 (0.18; 127.63)		1.40 (0.22; 9.09)	
		Adjusted	0.44 (0.19; 0.97)	*	0.20 (0.04; 0.97)	*	1.21 (0.32; 4.61)		0.26 (0.06; 1.11)		>999.9		0.94 (0.13; 7.11)	
Fruit & vegetable	at weekdays	Crude	1.14 (0.60; 2.15)		0.22 (0.08; 0.56)	**	0.42 (0.14; 1.22)		0.47 (0.21; 1.05)		0.92 (0.19; 4.45)		0.10 (0.02; 0.55)	*
(ref. bottom tertile)		Adjusted	1.14 (0.60; 2.17)		0.22 (0.08; 0.58)	**	0.43 (0.14; 1.29)		0.45 (0.19; 0.98)	*	0.34 (0.05; 2.35)		0.08 (0.01; 0.46)	**
	at weekend	Crude	1.98 (0.88; 4.47)		0.54 (0.17; 1.67)		1.40 (0.40; 4.92)		1.77 (0.53; 5.92)		0.02 (<0.01; 0.75)	*	0.17 (0.02; 1.43)	
		Adjusted	2.05 (0.90; 4.68)		0.58 (0.18; 1.85)		1.17 (0.31; 4.42)		1.92 (0.53; 7.00)		<0.001		0.26 (0.03; 2.55)	
Wholemeal food	at weekdays	Crude	2.50 (1.30; 4.82)	**	0.96 (0.36; 2.52)		0.66 (0.26; 1.71)		0.59 (0.26; 1.34)		0.80 (0.15; 4.38)		4.76 (0.73; 31.21)	
(ref. bottom tertile)		Adjusted	2.56 (1.31; 5.00)	**	0.88 (0.32; 2.40)		0.65 (0.25; 1.71)		0.56 (0.24; 1.32)		0.62 (0.11; 3.64)		4.41 (0.66; 29.44)	
	at weekend	Crude	2.96 (1.17; 7.46)	*	0.41 (0.12; 1.39)		0.38 (0.12; 1.26)		0.48 (0.16; 1.45)		0.03 (<0.01; 2.29)		1.04 (0.14; 7.67)	
		Adjusted	2.83 (1.10; 7.27)	*	0.37 (0.11; 1.28)		0.37 (0.11; 1.28)		0.34 (0.10; 1.13)		<0.001		1.41 (0.16; 12.47)	
Fruit &vegetable juices (ref. bottom tertile)	at weekdays	Crude	0.72 (0.37; 1.37)		1.05 (0.44; 2.55)		1.03 (0.43; 2.47)		1.77 (0.81; 3.87)		1.48 (0.30; 7.21)		0.42 (0.10; 1.88)	
		Adjusted	0.75 (0.38; 1.47)		0.91 (0.36; 2.25)		0.97 (0.39; 2.43)		2.01 (0.90; 4.52)		0.92 (0.16; 5.49)		0.47 (0.09; 2.53)	
	at weekend	Crude	0.59 (0.24; 1.44)		0.79 (0.27; 2.29)		0.44 (0.13; 1.49)		0.95 (0.33; 2.73)		1.62 (0.08; 31.75)		0.54 (0.08; 3.44)	
		Adjusted	0.63 (0.25; 1.59)		0.74 (0.25; 2.23)		0.58 (0.16; 2.06)		0.91 (0.29; 2.83)		<0.001		0.46 (0.05; 4.08)	
Meat & meat products (ref. bottom tertile)	at weekdays	Crude	0.91 (0.47; 1.77)		1.37 (0.56; 3.36)		1.89 (0.72; 4.96)		2.38 (1.08; 5.25)	*	0.28 (0.04; 2.23)		0.49 (0.11; 2.19)	
		Adjusted	0.90 (0.46; 1.77)		1.30 (0.52; 3.25)		2.12 (0.78; 5.75)		2.29 (1.02; 5.17)	*	0.35 (0.04; 3.37)		0.42 (0.08; 2.26)	
	at weekend	Crude	1.14 (0.46; 2.84)		0.66 (0.20; 2.18)		0.51 (0.14; 1.85)		1.87 (0.62; 5.61)		<0.001		0.25 (0.02; 2.91)	
		Adjusted	1.07 (0.43; 2.67)		0.64 (0.19; 2.16)		0.44 (0.11; 1.71)		1.54 (0.48; 4.90)		<0.001		0.31 (0.02; 4.16)	

^a^ Odds ratio unadjusted ^b^ Odds ratio adjusted for age, education and place of residence; Statistically significant: * *p* < 0.05; ** *p* < 0.01 (Wald’s test).
